# A Novel Gene, *fudoh,* in the SCC*mec* Region Suppresses the Colony Spreading Ability and Virulence of *Staphylococcus aureus*

**DOI:** 10.1371/journal.pone.0003921

**Published:** 2008-12-11

**Authors:** Chikara Kaito, Yosuke Omae, Yasuhiko Matsumoto, Makiko Nagata, Hiroki Yamaguchi, Taiji Aoto, Teruyo Ito, Keiichi Hiramatsu, Kazuhisa Sekimizu

**Affiliations:** 1 Laboratory of Microbiology, Graduate School of Pharmaceutical Sciences, The University of Tokyo, Hongo, Bunkyo-ku, Tokyo, Japan; 2 Division of Hematology, Department of Internal Medicine, Nippon Medical School, Bunkyo-ku, Tokyo, Japan; 3 Department of Central Laboratory, Nippon Medical School, Bunkyo-ku, Tokyo, Japan; 4 Department of Infection Control Science, Faculty of Medicine, Juntendo University, Bunkyo-ku, Tokyo, Japan; Baylor College of Medicine, United States of America

## Abstract

*Staphylococcus aureus* colonies can spread on soft agar plates. We compared colony spreading of clinically isolated methicillin-sensitive *S. aureus* (MSSA) and methicillin-resistant *S. aureus* (MRSA). All MSSA strains showed colony spreading, but most MRSA strains (73%) carrying SCC*mec* type-II showed little colony spreading. Deletion of the entire SCC*mec* type-II region from these MRSA strains restored colony spreading. Introduction of a novel gene, *fudoh,* carried by SCC*mec* type-II into Newman strain suppressed colony spreading. MRSA strains with high spreading ability (27%) had no *fudoh* or a point-mutated *fudoh* that did not suppress colony spreading. The *fudoh*-transformed Newman strain had decreased exotoxin production and attenuated virulence in mice. Most community-acquired MRSA strains carried SCC*mec* type-IV, which does not include *fudoh,* and showed high colony spreading ability. These findings suggest that *fudoh* in the SCC*mec* type-II region suppresses colony spreading and exotoxin production, and is involved in *S. aureus* pathogenesis.

## Introduction

*Staphylococcus aureus* is a pathogenic bacterium that causes various diseases in humans. In the last few decades, the emergence of methicillin-resistant *S. aureus* (MRSA), vancomycin-intermediate *S. aureus*, vancomycin-resistant *S. aureus*, and community acquired methicillin-resistant *S. aureus* (CA-MRSA) has caused serious clinical problems [1], [2]. A better understanding of the virulence mechanism of the MRSA strains is important for establishing effective therapeutic strategies. Whether the virulence of MRSA is different from that of MSSA, however, is controversial [3], [4]. The virulence of CA-MRSA is higher than that of MRSA [5]–[7], a difference thought to be related to the production of Panton-Valentine leukocidin [8], [9] and phenol-soluble modulin (PSM) [10], but the regulatory mechanisms causing enhanced expressions of these toxins has not been identified.

*S. aureus* is a non-flagellated gram-positive bacterium that until recently was believed not to have the ability to translocate. We previously reported that *S. aureus* has the ability to spread on soft agar surfaces, which we termed “colony spreading” [11]. Because bacterial translocation, such as flagella-driven swimming, is considered a bacterial virulence mechanism [12]–[14], the spreading ability of *S. aureus* is suspected to have a role in its virulence. In this study, we examined the spreading ability of clinically isolated MSSA, MRSA, and CA-MRSA strains, and report a novel gene that affects both the colony spreading ability and virulence of *S. aureus*.

## Results

### Most MRSA strains have less colony spreading ability than MSSA strains

**Figure 1:**
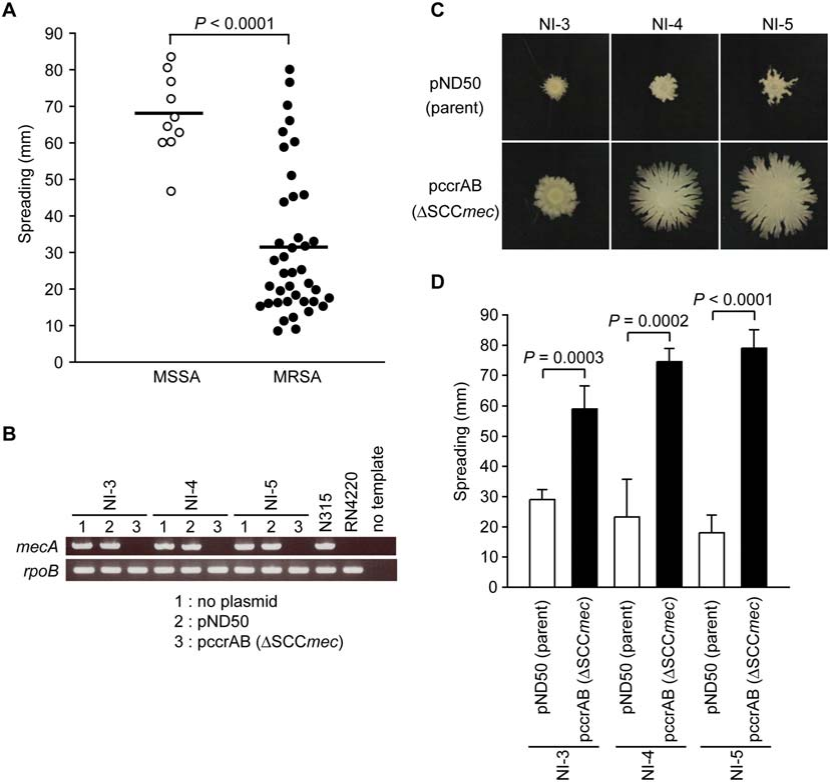
Deletion of the SCC*mec* region of the MRSA isolates increases colony spreading. (A) Comparison of colony spreading ability between MSSA and MRSA. Overnight cultures of clinical isolates of MSSA and MRSA were spotted onto soft agar plates and incubated for 10 h at 37°C. Two independent experiments with duplicates were performed and the mean halo diameters are presented as dots. The group means of MSSAs and MRSAs are presented as a bold bar. Statistical analysis was performed with Student's *t* test. (B) The *mecA* gene and the partial region of *rpoB* were amplified by PCR and electrophoresed and stained with ethidium bromide. The *rpoB* was used as control. (C) Overnight culture of NI-3, NI-4, and NI-5 harboring pND50 or pccrAB (ΔSCC*mec*) was spotted onto soft agar plates and incubated for 10 h. Representative images from three independent experiments are shown. (D) The halo diameter was measured and the means±standard deviations from three independent experiments are presented. Statistical analysis was performed with Student's *t* test.

We examined the colony spreading of 10 MSSA strains and 40 MRSA strains on soft agar plates. All MSSA strains showed colony spreading, and the average diameter of the spread after 10 h incubation was 68 mm. In contrast, the average diameter of the spread of the MRSA strains was 32 mm, which was significantly smaller than that of MSSA. The diameter of the spread of 73% of the MRSA strains was under 35 mm. The remaining MRSA strains showed colony spreading comparable to that of MSSA (Figure 1A). Therefore, we examined the reason for the decreased colony spreading of most of the MRSA strains, and the high colony spreading of some MRSA strains and all MSSA strains.

### Deletion of the SCC*mec* region restores the colony spreading ability of MRSA strains

One difference between MRSA and MSSA is the SCC*mec* region, which harbors the *mecA* gene encoding PBP2' (or PBP2a) that confers resistance against methicillin. We hypothesized that the SCC*mec* region was involved in the decreased colony spreading of most MRSA strains. To evaluate this possibility, we deleted the SCC*mec* region from the MRSA strains and examined colony spreading. MRSA strains NI-3, NI-4, and NI-5, which showed decreased colony spreading, were transformed with a plasmid carrying *ccrAB* genes, which encode the recombinases that catalyze precise excision of SCC*mec* from the chromosome. The deletion of SCC*mec* in the *ccrAB-*transformants was verified by a decrease in the methicillin minimum inhibitory concentration (MIC) values to 2 µg/ml, and by the absence of the *mecA* gene based on the results of polymerase chain reaction (PCR) experiments (Figure 1B). The SCC*mec*-deleted mutants showed higher colony spreading than their respective parent strains (Figure 1C and 1D). Expression of the *ccrAB* genes itself did not alter colony spreading of the RN4220 strain, a laboratory strain of *S. aureus* (Figure S1A and S1D). Therefore, we concluded that the colony spreading of MRSA strains is suppressed by a factor (factors) encoded by SCC*mec*.

### A newly identified gene, *fudoh,* which locates close to the *mecI* gene, suppresses the colony spreading of *S. aureus*

**Figure 2:**
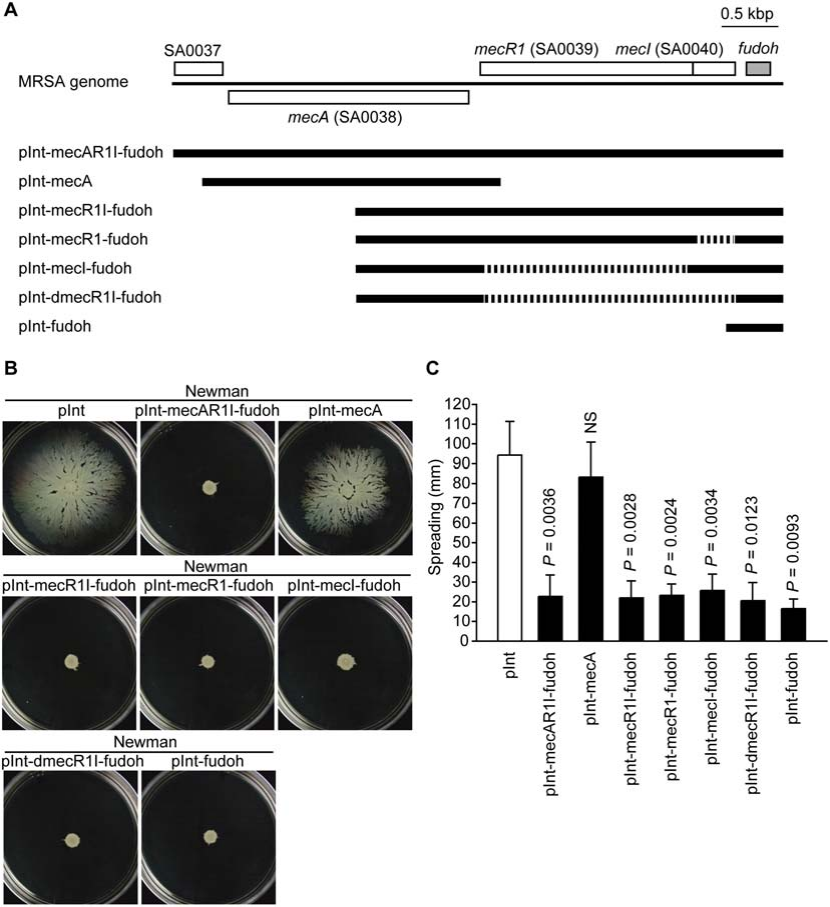
A novel gene, *fudoh,* in the *SCCmec* region suppresses the colony spreading ability of the Newman strain. (A) Schematic representation of the *mecA, mecR1, mecI,* and *fudoh* genes in the SCC*mec* region. MRSA chromosomal DNA is represented as a thin line, and the DNA fragment is represented by a thick line, and the deleted DNA region is represented by a dotted line. The boxes above the thin line indicate genes transcribed from left to right, whereas the box below the thin line indicates gene transcribed from right to left. Grey box represents the *fudoh* gene. (B) The Newman strain was transformed with the integration plasmids described in (A), and colony spreading was examined. The photograph was taken after 10 h incubation. (C) The means±standard deviations of the halo diameters of at least two independent experiments are presented. Statistical analysis was performed with Student’s *t* test. The *P-*values are versus pInt. NS, not significant (*P*>0.05).

To identify the gene in the SCC*mec* region that is responsible for suppressing the colony spreading of *S. aureus*, we integrated various flanking regions of the *mecA* gene of the SCC*mec* region (Figure 2A) into the chromosome of the Newman strain, a laboratory MSSA strain, using an integration vector by a single homologous recombination to examine the colony spreading of the recombinant strains. The Newman strain integrated with the *mecA-R1-I* gene complex and its surrounding regions (pInt-mecAR1I-fudoh) showed less colony spreading than the parent strain integrated with an empty vector (pInt) (Figure 2B and 2C). Thus, a gene (genes) in this region suppresses the colony spreading of *S. aureus*. To examine which subregion of the pInt-mecAR1I-fudoh was responsible for the suppression, we constructed integration plasmids harboring each subregion of the plasmid. The subregion containing the *mecA* gene (pInt-mecA) had little effect on colony spreading, but the subregion containing the *mecR1I* genes (pInt-mecR1I-fudoh) suppressed colony spreading. The vectors (pInt-mecI-fudoh, pInt-mecR1-fudoh, pInt-dmecR1I-fudoh) containing deletions of *mecR1*, *mecI,* or both from pInt-mecR1I-fudoh suppressed the colony spreading ability of the Newman strain (Figure 2B and 2C). Thus, the gene that suppresses colony spreading is neither the *mecR1* gene nor the *mecI* gene. We searched for open reading frames (ORFs) in the residual region and found an ORF encoding 70 amino acids downstream of the *mecI* gene (position 49366–49578 of the *S. aureus* N315 genome database [15], Figure 2A). The integration plasmid harboring this ORF (pInt-fudoh) suppressed colony spreading of the Newman strain (Figure 2B, 2C, and Movie S1), indicating that a single copy of the ORF suppresses colony spreading of *S. aureus*. The ORF has not been registered in the *S. aureus* N315 genome database, and its function is unknown. We named the gene *fudoh* (GenBank accession number AB442164), which means ‘non-motile’ in Japanese. A motif search against the *fudoh* gene product identified a transmembrane domain in amino acid residues 5 to 24, but no other motifs.

We searched the *fudoh* gene from SCC*mec* type-I to type-V sequences deposited on GenBank (type-I, AB033763; type-II, D86934; type-III, AB037671; type-IV, AJ810121; type-V, AB121219), and found that *fudoh* exists in SCC*mec* types-II and -III, but not in types-I, -IV, and -V in *S. aureus*. To verify whether the SCC*mec* type-III including *the fudoh* gene suppresses the colony spreading, we examined the colony spreading of the type-III SCC*mec*-deleted mutants of four clinical isolates 86/560, 86/961, 86/2652, and 85/3566 [16]. Deletion of the entire SCC*mec* type-III region from two of four strains, 86/961 and 85/3566, increased colony spreading (Figure S2), suggesting that *fudoh* in type-III SCC*mec* also has the function to suppress the colony spreading. Deletion of SCC*mec* from other two strains did not increase the colony spreading (data not shown), indicating that there are other mechanisms to suppress the colony spreading.

To exclude the possibility that integration of the vector into the Newman chromosome altered the gene expression and contributed to suppress colony spreading, we examined whether *fudoh* expression from a multicopy plasmid suppressed the colony spreading ability of *S. aureus*. Plasmid-induced expression of *fudoh* suppressed colony spreading of the Newman strain (Figure S1B). Therefore, the suppression of colony spreading by *fudoh* was not an artifact caused by integration of the vector into the chromosome. The *fudoh*-induced suppression of colony spreading was also observed in RN4220 strain (Figure S1C and S1D), suggesting that *fudoh*-induced suppression of colony spreading ability is a common mechanism in *S. aureus.* To address whether the differences of the colony spreading ability could be attributed to the differences of growth rates, we examined the growth curves of the *fudoh*-transformed Newman and the SCC*mec*-deleted mutants of NI-3, NI-4, and NI-5. There were no differences of growth curves between NI-3, NI-4, and the SCC*mec*-deleted mutants (Figure S3), indicating that the differences of the colony spreading cannot be explained by their growth rates. We found that the *fudoh*-transformed Newman showed slight longer lag period than the empty vector-transformed Newman, although the doubling time was both 30 min. The doubling time of the SCC*mec*-deleted mutant of NI-5 was 27 min, which was slight shorter than that of the parent strain, 33 min. These differences of growth could contribute to the differences of colony spreading ability.

### *fudoh* is mutated in highly spreading MRSA isolates

**Figure 3:**
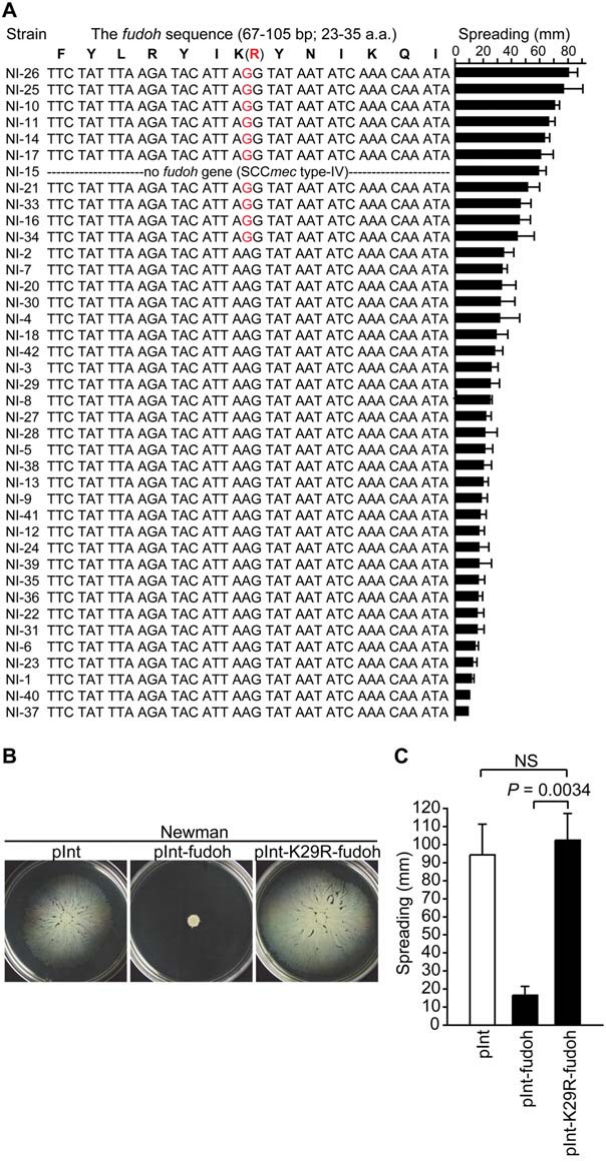
The *fudoh* gene is mutated in high-spreading MRSA strains. (A) The *fudoh* gene of 40 MRSA strains was sequenced. The sequences are organized according to the amount of colony spreading. The amounts of colony spreading are shown in the right bar graph from higher to lower values. The mutated nucleotide residues and the substituted amino acid residue are colored red. (B) The Newman strain was transformed with an integration plasmid harboring either the intact *fudoh* gene (pInt-fudoh) or a mutated *fudoh* gene (pInt-K29R-fudoh), and colony spreading was examined. The photograph was taken after 10 h incubation. (C) The means±standard deviations of the halo diameters from three independent experiments are presented.

Some MRSA strains showed high colony spreading ability that was comparable to that of the MSSA strains (Figure 1A). We hypothesized that *fudoh* may be mutated in the high-spreading MRSA strains. To test this notion, we sequenced the *fudoh* gene in all 40 MRSA strains. Ten MRSA strains that showed high colony spreading had a point mutation in the *fudoh* gene inducing a K29R amino acid substitution (Figure 3A). Another high-spreading MRSA strain, NI-15, did not contain the *fudoh* gene. There was no mutation in the *fudoh* genes of the remaining 29 MRSA strains (73%) with decreased colony spreading ability (spread diameters <35 mm/10 h) (Figure 3A). Multiplex-PCR identified the SCC*mec* of NI-15 as SCC*mec* type-IV [17], which lacks the *fudoh* gene, whereas most MRSA strains (37 strains) contained SCC*mec* type-II.

To determine whether the point-mutated *fudoh* gene loses the suppression activity against colony spreading, we examined colony spreading of Newman transformed with *fudoh* with a K29R point mutation. The K29R-mutated *fudoh* did not suppress colony spreading of the Newman strain (Figure 3B, 3C, and S1B). The loss of the suppression activity of the K29R-mutated *fudoh* was also observed in the RN4220 strain (Figure S1C and S1D). Thus, the K29R-mutated *fudoh* loses the ability to suppress colony spreading of *S. aureus*. The increased colony spreading of 11 MRSA strains can thus be explained by a mutation in, or absence of, the *fudoh* gene.

### The Newman strain transformed with the *fudoh* gene produces less exotoxin and has attenuated virulence in mice

**Figure 4:**
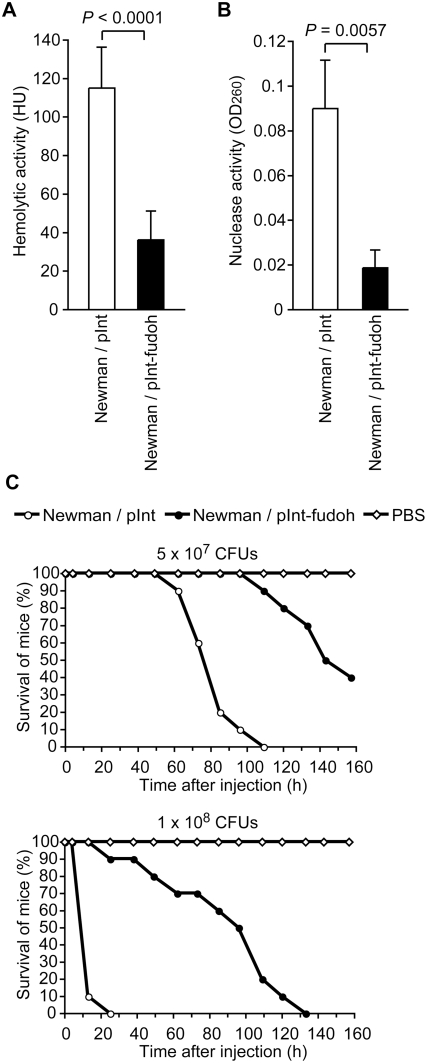
The *fudoh* gene decreases *S. aureus* exotoxin production and virulence in mice. (A) Hemolytic activities of culture supernatants of the Newman strains transformed with pInt or pInt-fudoh were measured using sheep erythrocytes. The data are means±standard deviations from three independent experiments. (B) Nuclease activities were measured using salmon sperm DNA. (C) CD-1 mice (n = 10) were injected intravenously with a diluted bacterial solution of Newman/pInt or Newman/pInt-fudoh. Statistical analysis was performed with the Kaplan-Meier test. The *P-*value between pInt and pInt-fudoh is less than 0.0001.

To determine whether the *fudoh* gene affects the virulence of *S. aureus*, we examined the exotoxin production of the Newman strain transformed with the *fudoh* gene. The Newman strain transformed with the *fudoh* gene produced less hemolysin and nuclease than the strain transformed with an empty vector (Figure 4A and 4B). These results indicate that the *fudoh* gene suppresses exotoxin production by *S. aureus*.

To examine the effect of *fudoh* on the virulence of *S. aureus* in animals, we studied the virulence of the Newman strain transformed with the *fudoh* gene in a murine model of systemic infection. Mice injected with the *fudoh*-transformed Newman strain lived longer than mice injected with the empty vector-transformed Newman (Figure 4C). Therefore, the *fudoh* gene suppressed *S. aureus* virulence in a mouse model.

### The colony spreading ability of CA-MRSA strains

**Figure 5:**
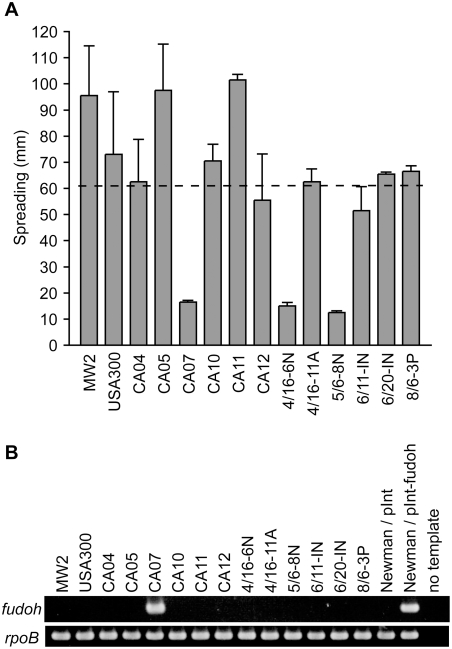
Colony spreading of CA-MRSA. (A) Overnight cultures of CA-MRSA strains were spotted onto soft agar plates and incubated for 10 h at 37°C. The dotted line indicates the averaged value. (B) The DNA fragments containing *fudoh* and the partial region of *rpoB* were amplified by PCR using the CA-MRSA genomes, Newman/pInt genome, or Newman/pInt-fudoh genome as the template. The amplified DNA was electrophoresed in 1% agarose gel and stained with ethidium bromide.

To gain further insight into the relation between the *fudoh* gene, colony spreading ability, and virulence, we examined colony spreading ability and the presence of the *fudoh* gene in CA-MRSA strains, which have high virulence and have been the recent cause of serious clinical problems. MW2 (USA400) was isolated in North Dakota in 1998, USA300 was isolated in San Francisco in 2000, and the others were isolated in Chicago between 1996 and 1999 [6], [18], [19]. The average diameter of the spread of these CA-MRSAs was 60 mm (Figure 5A), which was significantly greater than that of MRSAs (*P* = 0.0001). Amplification of the *fudoh* gene by PCR revealed that 13 of 14 tested strains (except for CA07) did not have the *fudoh* gene (Figure 5B). The result was consistent with reports that these strains (other than CA07) have SCC*mec* type IV [6], [18], [19], the type that does not contain the *fudoh* gene. Thus, most CA-MRSAs have increased colony spreading ability because they lack the *fudoh* gene. CA07 possessed the *fudoh* gene, and had decreased colony spreading, consistent with the idea that the *fudoh* gene suppresses *S. aureus* colony spreading. Strains 4/16-6N and 5/6-8N showed decreased colony spreading, despite their lack of the *fudoh* gene. These strains may have an unidentified mechanism that decreases colony spreading ability in the absence of the *fudoh* gene.

## Discussion

The findings of the present study revealed that the colony spreading ability of MSSAs is greater than that of most MRSAs. Furthermore, our data indicate that the *fudoh* gene in the SCC*mec* region decreases the colony spreading ability of MRSAs. *S*. *aureus* containing the *fudoh* gene also had decreased exotoxin production and decreased virulence in mice. This is the first observation that SCC*mec*, which carries the methicillin-resistance determinant, is involved in suppressing the colony spreading and virulence of *S. aureus.* Most MRSAs isolated in this study had SCC*mec* type-II, which is typically found in MRSA strains associated with hospitals or other health-care institutions (HA-MRSAs). Thus, it is considered that the MRSAs that acquired SCC*mec* type-II have reduced colony spreading and virulence, although they have also acquired resistance against all beta-lactam antibiotics. We further demonstrated that colony spreading was restored in MRSAs harboring the mutated *fudoh* gene in SCC*mec* type-II. The result indicates that the *fudoh* gene is not a constant suppressor of colony spreading and virulence of *S. aureus*, but also can function in restoring these abilities by its mutation.

CA-MRSA strains carrying SCC*mec* type-IV that includes no *fudoh* showed greater colony spreading than MRSAs carrying SCC*mec* type-II. CA-MRSAs cause more severe infections and produce higher amounts of exotoxins than HA-MRSAs [10]. Introduction of *fudoh* into the Newman strain decreased exotoxin production and colony spreading. These results suggest that the absence of *fudoh* in SCC*mec* type-IV is a plausible cause of the increased colony spreading, exotoxin production, and virulence of CA-MRSAs. Examination of SCC*mec* type-I to type-V sequences obtained from the DNA database suggest that *fudoh* exists in SCC*mec* types-II and -III, but not in types-I, -IV, and -V. Investigation of the relation between the existence of *fudoh* or the *fudoh* mutation and the clinical observations will contribute to a better understanding of biologic role of *fudoh* in SCC*mec* types-II and -III and to the development of more effective detection methods of highly virulent MRSAs and therapies against MRSA infections. SCC*mec* is not only present in *S. aureus*, but also in other staphylococcal species. *Staphylococcus epidermidis* RP62A has *fudoh* (ORF number, SERP2518) in its SCC*mec* region. *S. haemolyticus*, *S. hominis*, and *S. saprophyticus* are deduced to carry *fudoh* from the typing of their SCC*mec* [20], [21]. The *fudoh* gene has not been found in other chromosomal regions or in other organisms sequenced to date. As SCC*mec* is a foreign DNA introduced into *Staphylococcus* species [22], the coexistence of the *fudoh* and methicillin resistance genes in the same SCC*mec* might be a survival strategy of this foreign DNA to modulate virulence as well as methicillin resistance to increase the fitness of the host bacteria in the infected animal body. Such positive effect of the *fudoh* gene onto the fitness of bacteria has not been demonstrated yet in our study. It should be noted that the suppressing effect of *fudoh* onto virulence might be meaningful itself if the bacteria will not kill host animals by modulating their virulence to coexist with host animals. This possibility should be examined using other infection models. Our preliminary results from a microarray analysis suggested that *fudoh* affects the expression of many other genes. Elucidating the mechanisms underlying how the *fudoh* gene product, presumably a membrane-associated 70-aminoacid peptide, decreases *S. aureus* colony spreading on soft agar plates, and exotoxin production and virulence in mice will enhance our understanding of the interrelationship between foreign DNA, host bacteria, and an infected animal.

## Materials and Methods

### Bacterial strains and growth conditions

The JM109 strain of *E. coli* was used as a host for pND50, pInt, and their derivatives. *E. coli* strains transformed with the plasmids were cultured at 37°C in Luria-Bertani broth containing 25 µg/ml chloramphenicol. *S. aureus* strains were aerobically cultured in tryptic soy broth at 37°C, and 12.5 µg/ml chloramphenicol was added to the medium to maintain the plasmids. Clinical isolates of MSSA and MRSA strains were obtained from Nippon Medical School. These strains were streaked on mannitol sodium chloride plates (Eiken Chemical Inc., Tokyo, Japan) and their utilization of mannitol and high-salt resistance were confirmed. For MRSA strains, MIC values against methicillin were examined and resistance to methicillin was confirmed. Details of the bacterial strains and plasmids used in this study are shown in Table S1.

### Colony spreading assay

Tryptic soy broth (Becton, Sparks, MD) supplemented with 0.24% agar (Nacalai Tesque Inc., Kyoto, Japan) was autoclaved. Sterile medium (50 ml) was poured onto a plate (150 mm diameter, FALCON 351058, Becton Dickinson Labware, NJ). Plates were dried in a safety cabinet for 20 min before inoculation with bacteria. Overnight cultures of *S. aureus* (2 µl) were spotted onto the center of the plates using a P-20 Pipetteman (Gilson S. A. S., Villiers-le-Bel, France). After inoculation, the plates were dried in a safety cabinet for 15 min and incubated at 37°C for 10 h. Photographs were taken using a FinePix S9000 digital camera (Fuji Photo Film Co. Ltd., Tokyo, Japan).

### DNA manipulation

Transformation of *E. coli*, extraction of plasmid DNA from *E. coli*, PCR, and Southern blot analyses were performed as previously reported [23]. Extraction of genomic DNA from *S. aureus* was performed using a QIAamp DNA Blood Kit (Qiagen Sciences, Germantown, MD). *S. aureus* was transformed with plasmid DNA by electroporation [24]. Primers used in this study are listed in Table S2.

### Construction of SCC*mec* deletion mutants

The deletion of the SCC*mec* region was performed according to the method of Katayama *et. al.* [25]. The *ccrAB* genes were amplified by PCR using oligonucleotide primers FccrAB and RccrAB and the N315 genome as the template. The amplified fragments were inserted into pND50, resulting in pccrAB. The RN4220 strain was transformed with pccrAB by electroporation and the plasmids were extracted from colonies resistant to chloramphenicol. NI-3, NI-4, and NI-5 strains were transformed with the plasmid and strains resistant to chloramphenicol were obtained. Chloramphenicol-resistant colonies were inoculated into tryptic soy broth and cultured overnight. A 5-µl aliquot of overnight culture was inoculated into 5 ml of tryptic soy broth and cultured overnight. The inoculation was repeated. The overnight culture was plated onto tryptic soy agar and a single colony was isolated. The methicillin MIC of each strain was examined and methicillin-sensitive strains (2 µg/ml) were obtained. Frequencies of methicillin-sensitive strains from NI-3, NI-4, and NI-5 harboring pccrAB were 100%, 71%, and 100%, respectively. The *mecA* gene was amplified by PCR using genomes of each strain and oligonucleotide primers FmecA and RmecA.

### Construction of the Newman strain harboring the SCC*mec* region

The 717-bp of genomic region of RN4220 (41282–41998 in the NCTC8325 genome database) was amplified by PCR using primers Int-F and Int-R and inserted into the *Xba*I and *Hind*III sites of pCK20 [26], resulting in pInt, which is able to integrate into the *S. aureus* chromosome by a single homologous recombination. SCC*mec* regions were amplified by PCR using N315 genome as the template and inserted into the *Sma*I site of pInt, resulting in various plasmids as illustrated in Figure 2A. To construct pInt-mecR1-fudoh, pInt-mecI-fudoh, and pInt-dmecR1I-fudoh, the DNA fragments were amplified by PCR using pInt-mecR1I-fudoh as the template, and self-ligated. RN4220 strains were transformed with plasmids and colonies resistant to chloramphenicol were isolated. The chromosomal region harboring the plasmid was transferred to the Newman strain by phage 80 alpha transduction [27]. Integration of the SCC*mec* region into the desired chromosomal locus was confirmed by Southern blot analysis using the PCR-amplified region of SCC*mec* and pInt as probes. To express the *fudoh* gene from a multicopy plasmid, SCC*mec* regions were inserted into the *Sma*I site of pND50.

### Typing of the SCC*mec* region of clinical isolates

Multiplex PCRs were performed to identify the SCC*mec* types according to the established method [28]. Primer sets M-PCR1 and M-PCR2 were used. NI-7, NI-22, NI-36, and NI-38 were *ccrC* positive. NI-13 and NI-14 were not typed because DNA fragments were not amplified using M-PCR1 and M-PCR2.

### Mouse infection experiment

Bacterial suspensions (100 µl) were injected into the tail vein of 8-wk-old female CD-1 mice (Charles River Laboratories, Kanagawa, Japan) [29]. After the injection, mouse survival was monitored. All mouse protocols were reviewed by the Animal Use Committee at the Graduate School of Pharmaceutical Science at the University of Tokyo.

## Supporting Information

Movie S1 Time-lapse imaging of the colony spreading of the *fudoh*-transformed Newman. The colony spreading ability of the Newman strain transformed with an empty vector (pInt, left panel) or the integration plasmid harboring the intact *fudoh* gene (pInt-fudoh, right panel) was examined using time-lapse imaging. Ten hours observation was shortened to 20 seconds.
(3.31 MB MOV)

Figure S1 Plasmid-induced expression of *fudoh* suppresses colony spreading in Newman and RN4220. (A) Overnight culture of RN4220 harboring pND50 or pccrAB was spotted onto soft agar plates and incubated for 10 h. (B) The Newman strain was transformed with the plasmids described in Table S1, and colony spreading was examined. The means±standard deviations of the halo diameters of at least two independent experiments are presented. Statistical analysis was performed with Student's *t* test. The *P*-values are versus pND50. NS, not significant (*P*>0.05). (C) Overnight culture of RN4220 harboring pND50, pfudoh, or pK29R-fudoh was spotted onto soft agar plates and incubated for 10 h. The *P*-values are versus pND50. (D) Representative images of experiment (A) and (C) are presented.
(3.66 MB TIF)

Figure S2 Deletion of the SCC*mec* type-III region from MRSA 86/961 and 85/3566 increases colony spreading. (A) The *mecA*, the *fudoh*, and the partial region of *rpoB* were amplified by PCR and electrophoresed and stained with ethidium bromide. The *rpoB* was used as control. (B) Overnight cultures of 86/961 and 85/3566 harboring pND50 or pccrAB (ΔSCC*mec*) were spotted onto soft agar plates and incubated for 10 h. Representative images from three independent experiments are shown. (C) The halo diameter was measured and the means±standard deviations from three independent experiments are presented. Statistical analysis was performed with Student's *t* test.
(10.94 MB TIF)

Figure S3 Growth curves of the *fudoh*-transformed Newman strain and the SCC*mec*-deleted mutants of clinical isolates. Overnight cultures of *S. aureus* strains were inoculated with 100-fold dilution into fresh tryptic soy broth and incubated at 37°C with shaking. OD_600_ was measured. (A), Newman; (B), NI-3; (C), NI-4; (D), NI-5.
(0.49 MB TIF)

Table S1 A list of bacterial strains and plasmids used.
(0.18 MB DOC)

Table S2 PCR primers used in this study.
(0.06 MB DOC)

## References

[1] Hiramatsu K (2001). Vancomycin-resistant Staphylococcus aureus: a new model of antibiotic resistance.. Lancet Infect Dis.

[2] Chambers HF (2005). Community-associated MRSA–resistance and virulence converge.. N Engl J Med.

[3] Rozgonyi F, Kocsis E, Kristof K, Nagy K (2007). Is MRSA more virulent than MSSA?. Clin Microbiol Infect.

[4] Mizobuchi S, Minami J, Jin F, Matsushita O, Okabe A (1994). Comparison of the virulence of methicillin-resistant and methicillin-sensitive Staphylococcus aureus.. Microbiol Immunol.

[5] Voyich JM, Braughton KR, Sturdevant DE, Whitney AR, Said-Salim B (2005). Insights into mechanisms used by Staphylococcus aureus to avoid destruction by human neutrophils.. J Immunol.

[6] Diep BA, Gill SR, Chang RF, Phan TH, Chen JH (2006). Complete genome sequence of USA300, an epidemic clone of community-acquired meticillin-resistant Staphylococcus aureus.. Lancet.

[7] Baba T, Takeuchi F, Kuroda M, Yuzawa H, Aoki K (2002). Genome and virulence determinants of high virulence community-acquired MRSA.. Lancet.

[8] Labandeira-Rey M, Couzon F, Boisset S, Brown EL, Bes M (2007). Staphylococcus aureus Panton-Valentine leukocidin causes necrotizing pneumonia.. Science.

[9] Voyich JM, Otto M, Mathema B, Braughton KR, Whitney AR (2006). Is Panton-Valentine leukocidin the major virulence determinant in community-associated methicillin-resistant Staphylococcus aureus disease?. J Infect Dis.

[10] Wang R, Braughton KR, Kretschmer D, Bach TH, Queck SY (2007). Identification of novel cytolytic peptides as key virulence determinants for community-associated MRSA.. Nat Med.

[11] Kaito C, Sekimizu K (2007). Colony spreading in Staphylococcus aureus.. J Bacteriol.

[12] Josenhans C, Suerbaum S (2002). The role of motility as a virulence factor in bacteria.. Int J Med Microbiol.

[13] Krukonis ES, DiRita VJ (2003). From motility to virulence: Sensing and responding to environmental signals in Vibrio cholerae.. Curr Opin Microbiol.

[14] Cossart P, Sansonetti PJ (2004). Bacterial invasion: the paradigms of enteroinvasive pathogens.. Science.

[15] Kuroda M, Ohta T, Uchiyama I, Baba T, Yuzawa H (2001). Whole genome sequencing of meticillin-resistant Staphylococcus aureus.. Lancet.

[16] Suzuki E, Kuwahara-Arai K, Richardson JF, Hiramatsu K (1993). Distribution of mec regulator genes in methicillin-resistant Staphylococcus clinical strains.. Antimicrob Agents Chemother.

[17] Hiramatsu K, Cui L, Kuroda M, Ito T (2001). The emergence and evolution of methicillin-resistant Staphylococcus aureus.. Trends Microbiol.

[18] Daum RS, Ito T, Hiramatsu K, Hussain F, Mongkolrattanothai K (2002). A novel methicillin-resistance cassette in community-acquired methicillin-resistant Staphylococcus aureus isolates of diverse genetic backgrounds.. J Infect Dis.

[19] Naimi TS, LeDell KH, Boxrud DJ, Groom AV, Steward CD (2001). Epidemiology and clonality of community-acquired methicillin-resistant Staphylococcus aureus in Minnesota, 1996-1998.. Clin Infect Dis.

[20] Hanssen AM, Sollid JU (2007). Multiple staphylococcal cassette chromosomes and allelic variants of cassette chromosome recombinases in Staphylococcus aureus and coagulase-negative staphylococci from Norway.. Antimicrob Agents Chemother.

[21] Higashide M, Kuroda M, Omura CT, Kumano M, Ohkawa S (2008). Methicillin-resistant Staphylococcus saprophyticus isolates carrying staphylococcal cassette chromosome mec have emerged in urogenital tract infections.. Antimicrob Agents Chemother.

[22] Noto MJ, Kreiswirth BN, Monk AB, Archer GL (2008). Gene acquisition at the insertion site for SCCmec, the genomic island conferring methicillin resistance in Staphylococcus aureus.. J Bacteriol.

[23] Sambrook J, Fritsch EF, Maniatis T (1989). Molecular cloning : a laboratory manual..

[24] Kaito C, Kurokawa K, Matsumoto Y, Terao Y, Kawabata S (2005). Silkworm pathogenic bacteria infection model for identification of novel virulence genes.. Mol Microbiol.

[25] Katayama Y, Ito T, Hiramatsu K (2000). A new class of genetic element, staphylococcus cassette chromosome mec, encodes methicillin resistance in Staphylococcus aureus.. Antimicrob Agents Chemother.

[26] Ichihashi N, Kurokawa K, Matsuo M, Kaito C, Sekimizu K (2003). Inhibitory effects of basic or neutral phospholipid on acidic phospholipid-mediated dissociation of adenine nucleotide bound to DnaA protein, the initiator of chromosomal DNA replication.. J Biol Chem.

[27] Novick RP (1991). Genetic systems in staphylococci.. Methods Enzymol.

[28] Kondo Y, Ito T, Ma XX, Watanabe S, Kreiswirth BN (2007). Combination of multiplex PCRs for staphylococcal cassette chromosome mec type assignment: rapid identification system for mec, ccr, and major differences in junkyard regions.. Antimicrob Agents Chemother.

[29] Kaito C, Morishita D, Matsumoto Y, Kurokawa K, Sekimizu K (2006). Novel DNA binding protein SarZ contributes to virulence in Staphylococcus aureus.. Mol Microbiol.

