# Supplementary figures and images for: A Novel Gene, fudoh, in the SCCmec Region Suppresses the Colony Spreading Ability and Virulence of Staphylococcus aureus
Source: PLoS One. 2008 Dec 11;3(12):e3921. doi: 10.1371/journal.pone.0003921 (PMC2593785; doi:10.1371/journal.pone.0003921)

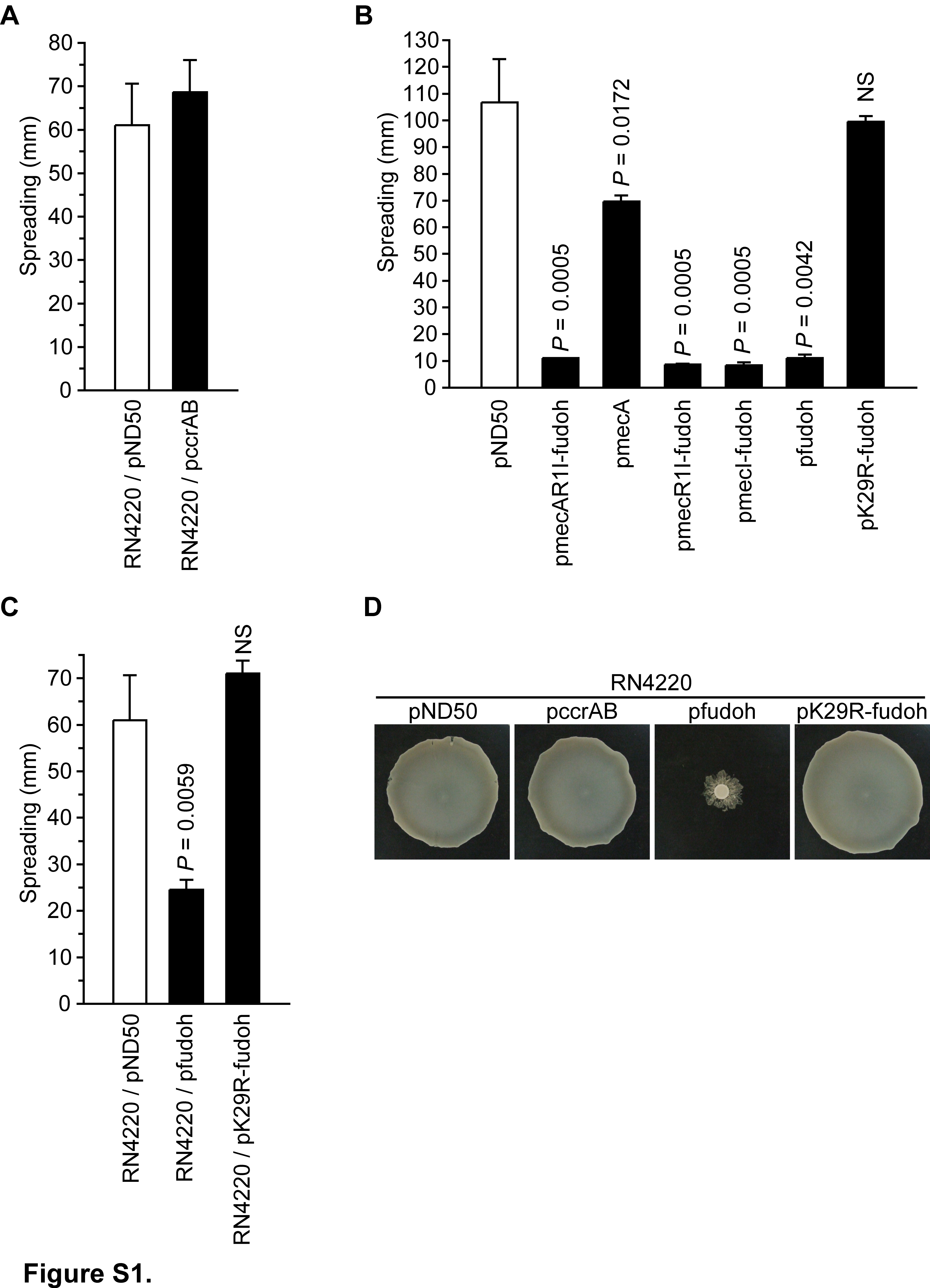

Supplement: Figure S1 — Plasmid-induced expression of fudoh suppresses colony spreading in Newman and RN4220. (A) Overnight culture of RN4220 harboring pND50 or pccrAB was spotted onto soft agar plates and incubated for 10 h. (B) The Newman strain was transformed with the plasmids described in Table S1, and colony spreading was examined. The means±standard deviations of the halo diameters of at least two independent experiments are presented. Statistical analysis was performed with Student's t test. The P-values are versus pND50. NS, not significant (P>0.05). (C) Overnight culture of RN4220 harboring pND50, pfudoh, or pK29R-fudoh was spotted onto soft agar plates and incubated for 10 h. The P-values are versus pND50. (D) Representative images of experiment (A) and (C) are presented. (3.66 MB TIF) [file pone.0003921.s002.tif]

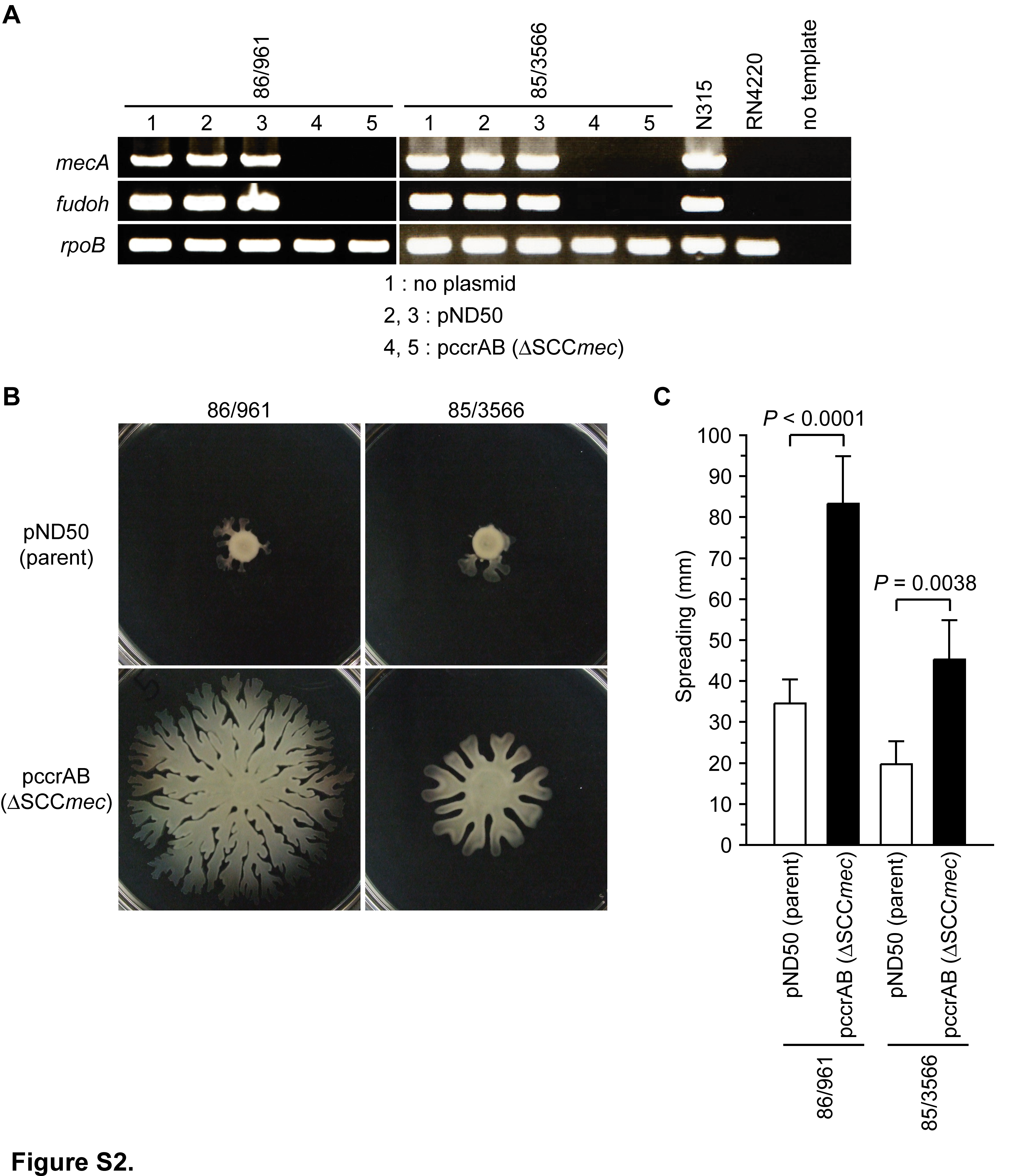

Supplement: Figure S2 — Deletion of the SCCmec type-III region from MRSA 86/961 and 85/3566 increases colony spreading. (A) The mecA, the fudoh, and the partial region of rpoB were amplified by PCR and electrophoresed and stained with ethidium bromide. The rpoB was used as control. (B) Overnight cultures of 86/961 and 85/3566 harboring pND50 or pccrAB (ΔSCCmec) were spotted onto soft agar plates and incubated for 10 h. Representative images from three independent experiments are shown. (C) The halo diameter was measured and the means±standard deviations from three independent experiments are presented. Statistical analysis was performed with Student's t test. (10.94 MB TIF) [file pone.0003921.s003.tif]

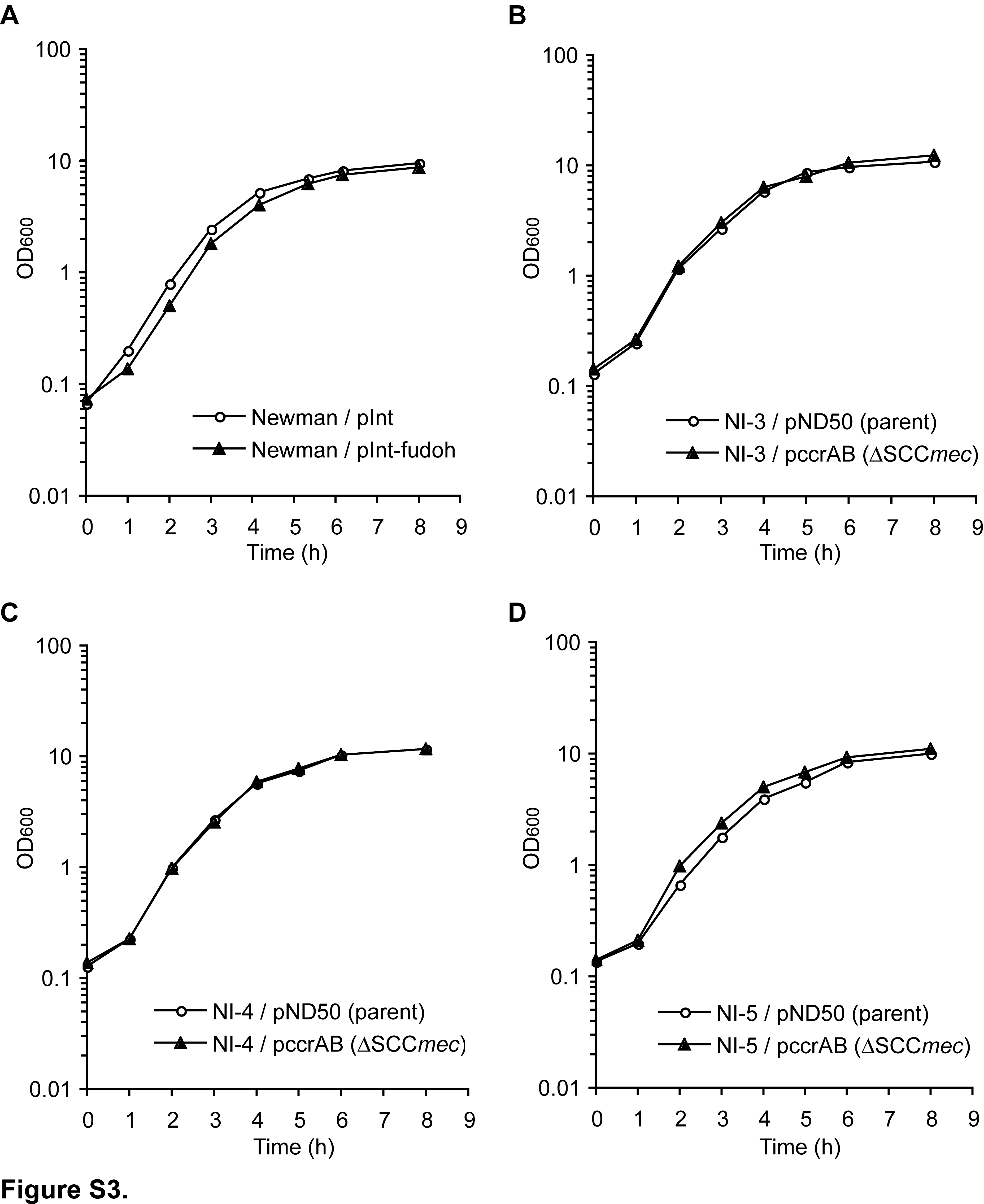

Supplement: Figure S3 — Growth curves of the fudoh-transformed Newman strain and the SCCmec-deleted mutants of clinical isolates. Overnight cultures of S. aureus strains were inoculated with 100-fold dilution into fresh tryptic soy broth and incubated at 37°C with shaking. OD600 was measured. (A), Newman; (B), NI-3; (C), NI-4; (D), NI-5. (0.49 MB TIF) [file pone.0003921.s004.tif]
