# Supplementary material for: A Novel Gene, fudoh, in the SCCmec Region Suppresses the Colony Spreading Ability and Virulence of Staphylococcus aureus
Source: PLoS One. 2008 Dec 11;3(12):e3921. doi: 10.1371/journal.pone.0003921 (PMC2593785; doi:10.1371/journal.pone.0003921)
Supplement: Table S1 — A list of bacterial strains and plasmids used. (0.18 MB DOC) [file pone.0003921.s005.doc]

**Table S1. A list of bacterial strains and plasmids used.**

| Strain or plasmid | Genotypes or characteristics^a^ | Source or reference |
| --- | --- | --- |
| Strains |  |  |
| *S. aureus* |  |  |
| RN4220 | NCTC8325-4, restriction mutant | [1] |
| Newman | Laboratory strain, High level of clumping factor | [2] |
| N315 | methicillin resistant | [3] |
| NI-1~NI-18 | Clinical isolates, methicillin resistant | This study |
| NI-20~NI-31 | Clinical isolates, methicillin resistant | This study |
| NI-33~NI-42 | Clinical isolates, methicillin resistant | This study |
| NIS-1~NIS-10 | Clinical isolates, methicilin sensitive | This study |
| 86/560 | methicillin resistant, type-III SCC*mec* | [4] |
| 86/961 | methicillin resistant, type-III SCC*mec* | [4] |
| 86/2652 | methicillin resistant, type-III SCC*mec* | [4] |
| 85/3566 | methicillin resistant, type-III SCC*mec* | [4] |
| MW2 | Community acquired MRSA, USA400 | [5] |
| USA300 | Community acquired MRSA | [6] |
| CA04 | Community acquired MRSA | [7] |
| CA05 | Community acquired MRSA | [7] |
| CA07 | Community acquired MRSA | [7] |
| CA10 | Community acquired MRSA | [7] |
| CA11 | Community acquired MRSA | [7] |
| CA12 | Community acquired MRSA | [7] |
| 4/16-6N | Community acquired MRSA | [7] |
| 4/16-11A | Community acquired MRSA | [7] |
| 5/6-8N | Community acquired MRSA | [7] |
| 6/11-IN | Community acquired MRSA | [7] |
| 6/20-IN | Community acquired MRSA | [7] |
| 8/6-3P | Community acquired MRSA | [7] |
|  |  |  |
| E. coli |  |  |
| JM109 | General purpose host strain for cloning | Takara Bio |
|  |  |  |
| Plasmids |  |  |
| pND50 | *E. coli*-*S. aureus* shuttle vector; Cm^r^ | [8] |
| pccrAB | pND50 with intact *ccrAB* from N315 | This study |
| pCK20 | *E. coli* vector; Cm^r^ | [9] |
| pInt | pCK20 with partial genomic region from RN4220 | This study |
| pInt-mecAR1I-fudoh | pInt with *mecAR1I* and *fudoh* from N315 | This study |
| pInt-mecA | pInt with *mecA* from N315 | This study |
| pInt-mecR1I-fudoh | pInt with *mecR1I* and *fudoh* from N315 | This study |
| pInt-mecR1-fudoh | pInt with *mecR1* and *fudoh* from N315 | This study |
| pInt-mecI-fudoh | pInt with *mecI* and *fudoh* from N315 | This study |
| pInt-dmecR1I-fudoh | pInt mecR1I-fudoh without *mecR1I* ORFs | This study |
| pInt-fudoh | pInt with *fudoh* from N315 | This study |
| pInt-K29R-fudoh | pInt with *fudoh* from NI-25 | This study |
| pmecAR1I-fudoh | pND50 with *mecAR1I* and *fudoh* from N315 | This study |
| pmecA | pND50 with *mecA* from N315 | This study |
| pmecR1I-fudoh | pND50 with *mecR1I* and *fudoh* from N315 | This study |
| pmecI-fudoh | pND50 with *mecI* and *fudoh* from N315 | This study |
| pfudoh | pND50 with *fudoh* from N315 | This study |
| pK29R-fudoh | pND50 with *fudoh* from NI-25 | This study |

**a**. Cm, chloramphenicol

1. Peng HL, Novick RP, Kreiswirth B, Kornblum J, Schlievert P (1988) Cloning, characterization, and sequencing of an accessory gene regulator (agr) in Staphylococcus aureus. J Bacteriol 170: 4365-4372.

2. Duthie ES, Lorenz LL (1952) Staphylococcal coagulase; mode of action and antigenicity. J Gen Microbiol 6: 95-107.

3. Kuroda M, Ohta T, Uchiyama I, Baba T, Yuzawa H, et al. (2001) Whole genome sequencing of meticillin-resistant Staphylococcus aureus. Lancet 357: 1225-1240.

4. Suzuki E, Kuwahara-Arai K, Richardson JF, Hiramatsu K (1993) Distribution of mec regulator genes in methicillin-resistant Staphylococcus clinical strains. Antimicrob Agents Chemother 37: 1219-1226.

5. Naimi TS, LeDell KH, Boxrud DJ, Groom AV, Steward CD, et al. (2001) Epidemiology and clonality of community-acquired methicillin-resistant Staphylococcus aureus in Minnesota, 1996-1998. Clin Infect Dis 33: 990-996.

6. Diep BA, Gill SR, Chang RF, Phan TH, Chen JH, et al. (2006) Complete genome sequence of USA300, an epidemic clone of community-acquired meticillin-resistant Staphylococcus aureus. Lancet 367: 731-739.

7. Daum RS, Ito T, Hiramatsu K, Hussain F, Mongkolrattanothai K, et al. (2002) A novel methicillin-resistance cassette in community-acquired methicillin-resistant Staphylococcus aureus isolates of diverse genetic backgrounds. J Infect Dis 186: 1344-1347.

8. Matsuo M, Kurokawa K, Nishida S, Li Y, Takimura H, et al. (2003) Isolation and mutation site determination of the temperature-sensitive murB mutants of Staphylococcus aureus. FEMS Microbiol Lett 222: 107-113.

9. Ichihashi N, Kurokawa K, Matsuo M, Kaito C, Sekimizu K (2003) Inhibitory effects of basic or neutral phospholipid on acidic phospholipid-mediated dissociation of adenine nucleotide bound to DnaA protein, the initiator of chromosomal DNA replication. J Biol Chem 278: 28778-28786.
