# Supplementary material for: A Novel Gene, fudoh, in the SCCmec Region Suppresses the Colony Spreading Ability and Virulence of Staphylococcus aureus
Source: PLoS One. 2008 Dec 11;3(12):e3921. doi: 10.1371/journal.pone.0003921 (PMC2593785; doi:10.1371/journal.pone.0003921)
Supplement: Table S2 — PCR primers used in this study. (0.06 MB DOC) [file pone.0003921.s006.doc]

**Table S2. PCR primers used in this study.**

| Target | Primer | Sequence(5'-3') |
| --- | --- | --- |
| *ccrAB* | FccrAB | TCTTCTAGACCGTGAAGCATCAAACTGAA |
|  | RccrAB | GGTGGTACCCGAAATTCCGCCTATTTTGA |
| Genomic region for integration | Int-F | AAGAAGCTTTTACGCATCCAAACACTCC |
|  | Int-R | TCTTCTAGAGCATGCCGTACCAATACATTT |
| *mecAR1I-fudoh* | mecAR1I-F | GGAGGATCCTCCATAATTGCCTACCCCATA |
|  | mecAR1I-R | AAGAAGCTTCGTTACGGATTGCTTCACTGT |
| *mecA* | mecA-F | GGAGGATCCTCGTGTCAGATACATTTCGATTCA |
|  | mecA-R | AAGAAGCTTGTTGTAGCAGGAACACAAATGAATAAC |
| *mecR1I-fudoh* | mecR1I-F | GGAGGATCCAACGATTGTGACACGATAGCC |
|  | mecR1I-R | AAGAAGCTTTCCATAATTGCCTACCCCATA |
| *mecR1-fudoh* | dmecI-F | ATATATTGAATAAAAAATAAA |
|  | dmecI-R | TCATACGTTTTATTATCCATT |
| *mecI-fudoh* | dmecR1-F | CGAAGACAATGCGAATGGCGAAAAAGCACAACAAA |
|  | dmecR1-R | CACATATCGTGAGCAATGAACTGATTATACTTAAC |
| *fudoh* | fudoh-F | CAATTCACTTGTCTTAAACTTTGTAGAAAAAGAAG |
|  | fudoh-R | TATTTTATTTTCCATAATTGCCTACCCCATAAG |
| *fudoh* sequencing | S2 | CAATTCACTTGTCTTAAACTTTGTAGAAAAAGAAG |
|  | S3 | TATTTTATTTTCCATAATTGCCTACCCCATAAG |
| *rpoB* | rpoB-F | GAAGAATTCAATTGGTTTGATGATTGCT |
|  | rpoB-R | GGTGGTACCCCAAACAGATTCACCCCTCA |
